# Left Bundle Branch Area Pacing Compared to Right Ventricular Outflow Tract Septal Pacing: Mid-term Results and Learning Curve

**DOI:** 10.19102/icrm.2024.15123

**Published:** 2024-12-15

**Authors:** Javier Ramos-Maqueda, Mercedes Cabrera-Ramos, Jorge Melero-Polo, Isabel Montilla-Padilla, Adrián Riaño-Ondiviela, José Ramón Ruiz-Arroyo

**Affiliations:** 1 Arrhythmia Unit, Department of Cardiology, University Hospital Clinico Lozano Blesa, Zaragoza, Spain; 2 Health Investigation Institute Aragon, Zaragoza, Spain

**Keywords:** Left bundle branch pacing, physiological pacing, right ventricular outflow tract septal pacing

## Abstract

Our study evaluated the efficacy and feasibility of left bundle branch area pacing (LBBAP) compared to right ventricular outflow tract septal pacing (RVOSP). We conducted a prospective, single-center, observational study involving 200 consecutive patients who required pacemaker implantation. The patients were divided into two groups (LBBAP and RVOSP), with 100 patients in each group. We aimed to compare the safety and efficacy, as well as the procedure and fluoroscopy times, between the two groups. Additionally, we aimed to describe the learning curve for the LBBAP group. The success and acute complication rates were similar (*P* = .56 vs. *P* = .65). The procedure time was longer in the LBBAP group compared to the RVOSP group (18 [13–28] vs. 11 [7–17] min; *P* < .001), while the fluoroscopy time was shorter in the LBBAP group compared to the RVOSP group (2.8 [1.3–3.7] vs. 3.1 [2–5.9] min; *P* = .02). The paced QRS interval was narrower in the LBBAP group (123.77 ± 10.25 vs. 159.79 ± 17.0 ms; *P* = .001). There were no significant differences in pacing parameters like R-wave sensing (9.6 ± 5.2 vs. 9.1 ± 4.7 mV; *P* = .91), bipolar impedance (685.9 ± 151.8 vs. 686.5 ± 158.6 Ω; *P* = .98), or pacing threshold (0.70 ± 0.29 vs. 0.64 ± 0.26 V @ 0.4 ms; *P* = .63). In the LBBAP group, both the procedure time (12 [10.5–15] vs. 32 [28.5–38.5] min; *P* < .001) and the fluoroscopy time (2 [1–4.6] vs. 5.1 [3.4–12] min; *P* < .01) were shorter in the last quartile (Q4) compared to the first quartile (Q1). The procedure time was similar between LBBAP Q4 and RVOSP (12 [10.5–15] vs. 11 [7–17] min; *P* = .33). LBBAP is as safe as RVOSP and achieves a narrower paced QRS compared to RVOSP. After a rapid learning curve, a shorter fluoroscopy time and a similar procedure time can be achieved.

## Introduction

Conventional pacing from the right ventricle (RVP) has been an effective treatment for bradyarrhythmias for decades. Traditionally, right ventricular apical pacing (RVAP) has been the most common RVP technique. It is well known that it causes electrical and mechanical dyssynchrony, leading to high rates of atrial fibrillation (20%) and heart failure (up to 60%). Additionally, it can cause progressive left ventricular systolic dysfunction in 10%–20% of cases, with an associated increase in mortality.^1–5^ In this context, right ventricular outflow tract septal pacing (RVOSP) was studied as an alternative to improve the prognosis in patients with a high expected pacing burden (>40%). It resulted in reduced worsening of the left ventricular function in patients with previous left ventricular dysfunction compared to RVAP and is equally safe and feasible.^6^ However, its potential benefit for patients with preserved ventricular function remains unclear, with inconsistent results.^6,7^

His bundle pacing (HBP) emerged to avoid the harmful effects of RVP and has demonstrated improved prognostic outcomes compared to conventional pacing.^8^ The latest European Pacing Guidelines recommend HBP as an alternative for patients with a high expected pacing burden.^9^ However, its extension has been limited due to technical challenges and a high and unstable capture threshold.^10^

LBBAP overcomes the challenges associated with HBP. It is safe and feasible^11,12^ and has gained more recognition in recent years due to its reproducibility and the narrower paced QRS it achieves compared to RVP.

LBBAP has shown superiority over conventional (apical or septal) pacing in an observational registry^13^; however, no studies to date have compared it to RVOSP. Additionally, its learning curve has not been described in detail.

Reported LBBAP fluoroscopy times are generally higher or similar to those of RVP.^14^ However, in the latter, intracardiac electrograms are usually not available or used, and, therefore, fluoroscopy is needed for every step of the procedure.

Our objective was to evaluate the safety and feasibility of LBBAP compared to RVOSP and compare the procedure and fluoroscopy times and paced QRS duration at implantation and during a 6-month follow-up. We also aimed to describe the learning curve of this novel pacing technique.

## Methods

We conducted a prospective, observational, single-center study that included 200 consecutive patients referred for pacemaker implantation from December 2019 to December 2020. All patients provided informed consent for the procedure and participation in the study. All research was conducted in accordance with the principles of the Declaration of Helsinki.

### Patients and the study groups

This study enrolled 200 consecutive patients with a pacing indication due to sinus node disease (SND), atrioventricular block (AVB), or atrial fibrillation with rapid ventricular response and AV node ablation indication. Patients were excluded if they were <18 years of age, pregnant, or had a left ventricular ejection fraction of <50%.

The pacing strategies were determined by operators’ preference, and we defined two groups: the first one included the initial 100 patients who underwent RVOSP (December 2019–April 2020), while the second group included the subsequent 100 patients who underwent LBBAP (April 2020–December 2020). Physicians only performed RVOSP within the first 5 months and LBBAP during the subsequent 7 months.

We compared the success rate, acute complication rate, procedure and fluoroscopy times, paced QRS duration, and the following acute pacing parameters: R-wave sensing, bipolar impedance, and pacing threshold. Each patient underwent a follow-up visit 10 days after the procedure for surgical wound revision and another visit at 6 months later, where a control electrocardiogram (ECG) was performed to remeasure paced QRS duration. Additionally, all patients were monitored remotely.

All procedures were carried out by two experienced operators proficient in RVOSP and with prior experience in LBBAP (10 previous cases). Both operators have significant expertise in performing zero- or low-fluoroscopy cardiac ablations, adhering to the “as low as reasonably achievable” (ALARA) principle to minimize radiation exposure.

In the LBBAP group, we further subdivided patients into the first 25 patients (first quartile, Q1) and the last 25 patients (last quartile, Q4) to assess the learning curve and evaluate differences in success rates, complications, procedure time, and fluoroscopy time.

### Procedure

All procedures were performed under local anesthesia, with sedation administered only when necessary. Venous access was primarily obtained via left axillary vein puncture, guided by fluoroscopy as the first approach (venography-guided in challenging cases), with an alternative access route through the subclavian vein. If left access was unattainable, we proceeded with right-sided access. We used the Lab System (Boston Scientific, Marlborough, MA, USA) polygraph to record a 12-lead ECG before and after the procedure. The procedure time in our study was defined as the duration of ventricular lead fixation, starting from the moment the lead crosses the sheath until its final position, as implantation of the atrial lead could confound the comparison between LBBAP and RVOSP. The fluoroscopy time was defined as fluoroscopy used during ventricular lead fixation.

In the RVOSP group, we employed a 58-cm bipolar active fixation electrode with steroid liberation (Medtronic CapsureFix 5076; Medtronic Inc., Minneapolis, MN, USA). The wire was manually preformed to reach the RVOSP position.^15^ We confirmed the lead position through fluoroscopy and 12-lead ECG. Regarding fluoroscopy, the posteroanterior and right anterior oblique projections were used to confirm the lead position in the vertical plane, with the lower border of the right ventricular outflow tract being defined as a line drawn perpendicular to the superior mitral annulus and the pulmonary valve serving as the upper border. Septal and free wall sites were determined by a leftward or rightward orientation, respectively, of the lead tip in the left anterior oblique view.

The paced QRS morphology in the 12-lead ECG should display a left bundle branch (LBB) block (LBBB) pattern with an inferior axis.^16^

LBBAP was performed using the SelectSecure pacing lead (Model 3830, 69 cm; Medtronic Inc.) with either a fixed or deflectable sheath (C315HIS and C304; Medtronic). Initially, His mapping was performed along the septum, and, once the His bundle signal was identified, the position of the sheath was marked in the right anterior oblique view on the fluoroscopy screen. Subsequently, the system was moved 1–2 cm anteriorly and inferiorly, following an imaginary line connecting the His and right ventricular apex, looking for a “W pattern” in lead V1 in the ECG. Upon locating this pattern, the sheath was rotated counterclockwise to ensure the electrode remained perpendicular to the septum, securing it properly for penetration. After 3–4 rapid clockwise rotations, and with subsequent repetitions, as the electrode penetrated the septum, a current of injury in the intracavitary electrogram was observed, and the “W” notch in V1 transitioned into a right bundle branch block (RBBB) pattern.^17^ The final confirmation of the electrode’s advanced position over the sheath was conducted on the fluoroscopy screen. In instances of left ventricular perforation, the electrode was retracted and repositioned in an alternate location.

### Success and complications definition

In the RVOSP group, success was determined by achieving a satisfactory radiological position in the three previously mentioned views, a consistent pattern in the surface ECG,^16^ and achieving a capture threshold of <2.0 V @ 0.4 ms with R-wave sensing of >4 mV. After five unsuccessful attempts, the approach was switched to RVAP.

Success in the LBBAP group was defined as achieving a pacing threshold of <1.5 V @ 0.4 ms, R-wave sensing of >4 mV, and confirmation of LBB capture using specific criteria.^17,18^ Specifically, this confirmation involved observing a combination of an RBBB pattern along with one of the following criteria: (1) the presence of left bundle potential, (2) abrupt shortening (>10 ms) of the left ventricular activation time (LVAT) with increasing voltage or maintenance of a short and constant LVAT both at low and high outputs, or (3) the presence of selective and non-selective LBBAP. After five unsuccessful attempts at LBBAP, the approach was switched to HBP. If HBP was also unsuccessful, then RVAP was performed.

The LBBAP group was further subdivided into two categories: (1) left ventricular septal pacing (LVSP) when an RBBB pattern was noticed but LBB capture criteria were not fulfilled and (2) LBB pacing (LBBP) if one of these criteria was fulfilled. We also classified LBBP cases into four subgroups with regard to the location of capture within the left ventricular conduction system, as described recently by Jastrzębski et al.^19^

Cases involve proximal LBB capture (LBB potential to the QRS interval value within the range of 35–25 ms and an inferior or intermediate QRS axis) and left bundle fascicular pacing (fascicular Purkinje potential to the QRS interval within the range of 24–0 ms or absence of a potential), with the latter further divided into left posterior fascicular pacing (LPFP) if a superior axis, left anterior fascicular pacing (LAFP) if an inferior axis, or left septal fascicular pacing (LSFP) if an intermediate QRS axis.

Complications related to the ventricular electrode were documented as acute complications occurring either during the procedure or during the following 24 h, as well as those occurring independently within the subsequent 6 months.

### Programming and follow-up

After the procedure, the device was programmed in the bipolar pacing mode and with algorithms to minimize ventricular pacing (AAI-DDD) in SND and paroxysmal AVB patients. For patients in the LBBAP group presenting with bundle branch block, AV delays were adjusted to achieve the shortest paced QRS duration, from the fusion with the intrinsic QRS. Automatic capture was activated, and all patients were provided with a remote monitoring device. An X-ray was performed 24 h after the procedure to rule out complications or electrode dislodgement. A follow-up appointment was scheduled for 6 months for all patients in both groups.

### Statistical analysis

All statistical analyses were performed using SPSS Statistics version 21.0 (IBM Corporation, Armonk, NY, USA). The results are reported as mean ± standard deviation (SD) or median (p25–p75) values. Clinical variables were compared using the chi-squared test or Fisher’s exact test for categorical variables and Student’s *t* test or the Mann–Whitney *U* test for continuous variables. *P* < .05 was considered statistically significant.

## Results

### Baseline clinical and procedural characteristics

Between December 2019 and December 2020, a total of 200 patients were enrolled, with 100 in the RVOSP group and 100 in the LBBAP group. The baseline characteristics revealed no differences **(Table 1)**. The median age of the cohort was 78.5 (73–82) years, with 63.5% being men. Pre-existing heart disease was present in 20% of patients. Regarding pacing indications, AVB was most common, accounting for 70.5% of cases, followed by SND in 25.5% and atrial fibrillation with rapid ventricular response in 4% of cases. Narrow QRS was present in 39.5% of cases and wide QRS was present in 60.5% (>120 ms), with RBBB being the most common conduction disorder (45%), followed by LBBB (14.5%) and intraventricular conduction disease (1%). A total of 158 patients (79%) received dual-chamber devices.

**Table 1:** Baseline Characteristics

	LBBAP (n = 100)	RVOSP (n = 100)	*P*
Male sex	60 (60)	67 (67)	.37
Age, years (Q1–Q3)	79 (71–81.75)	74 (78–83)	.94
Hypertension	62 (62)	68 (68)	.46
Diabetes mellitus	53 (53)	49 (49)	.78
Heart disease	24 (24)	16 (16)	.21
QRS characteristics			
Left bundle branch	11 (11)	18 (18)	.50
Right bundle branch	44 (44)	46 (46)	.56
Intraventricular conduction disease	0 (0)	2 (2)	.5
Narrow QRS	45 (45)	34 (34)	.4
Basal QRS duration (ms)	126.17 ± 28.3	130.21 ± 29.1	.27
Pacing indication			
SND	24 (24)	27 (27)	.74
Atrial fibrillation with rapid ventricular response	3 (3)	5 (5)	.44
AVB	73 (73)	68 (68)	.35
Pacing device			
Emergency implant	11 (11)	13 (13)	.56
Dual-chamber device	82 (82)	76 (76)	.38
Echocardiographic parameters			
LVEDD, mm	48.7 ± 6.6	49.2 ± 5.6	.73
LVEF, %	62.3 ± 7.1	60.8 ± 6.6	.69

*Abbreviations:* AVB, atrioventricular block; LBBAP, left bundle branch area pacing; LVEDD, left ventricular end-diastolic diameter; LVEF, left ventricular ejection fraction; RVOSP, right ventricular outflow tract septal pacing; SND, sinus node disease. Results are shown using mean ± standard deviation values or n (%) unless otherwise indicated. Heart disease includes coronary artery disease, cardiomyopathy, or valvular heart disease.

### Implant outcomes: success, complications, and pacing parameters

The success rates were similar in both groups, with 95% in the LBBAP group and 92% in the RVOSP group (*P* = .56). Acute complication rates were also similar, with both groups experiencing a 3% complication rate (*P* = .65). In the LBBAP group, complications included one pocket hematoma and two cases of interventricular septum perforation, without further repercussion (no clinical implications and the electrodes were successfully removed by simple traction and reimplanted in a different location). In both cases, the unipolar impedance dropped to 330 and 380 Ω, and the R-wave amplitude decreased to 0.9 and 1.1 mV, respectively. In the RVOSP group, there were two cases of pneumothorax and one case of pocket hematoma. No major complications, such as cardiac tamponade, stroke, or coronary artery injury, occurred in either group during the study. Right-sided access was required in five cases (three patients in the LBBAP group and two patients in the RVOSP group).

The procedure time was longer in the LBBAP group compared to the RVOSP group at 18 (13–28) versus 11 (7–17) min (*P* < .001) **(Table 2)**. The average number of ventricular lead fixation attempts was similar between the RVOSP group (1.6 ± 0.69) and the LBBAP group (1.7 ± 0.8).

**Table 2:** Pacing Parameters and Ventricular Electrode Outcomes

	LBBAP (n = 100)	RVOSP (n = 100)	*P*
R-wave, mV	9.6 ± 5.2	9.1 ± 4.7	.91
Pacing threshold, V @ 0.4 ms (acute)	0.70 ± 0.29	0.64 ± 0.26	.63
Pacing threshold, V @ 0.4 ms (6-month follow-up)	0.78 ± 0.23	0.63 ± 0.28	.56
Bipolar impedance, Ω	685.9 ± 151.8	686.5 ± 158.6	.98
Procedure time, min	18 (13–28)	11 (7–17)	<.001
Fluoroscopy time, min	2.8 (1.3–3.7)	3.1 (2–5.9)	.02
Paced QRS duration, ms	123.77 ± 10.25	159.79 ± 17.0	<.0001

*Abbreviations:* LBBAP, left bundle branch area pacing; RVOSP, right ventricular outflow tract septal pacing. Results are shown as mean ± standard deviation or median (Q1–Q3) values.

**Figure 1:**
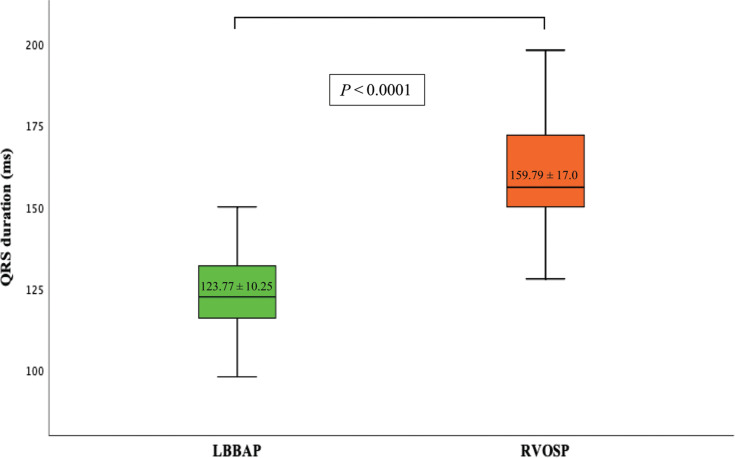
Comparison of paced QRS duration (ms) between left bundle branch area pacing and right ventricular outflow tract septal pacing. The results are shown as mean ± standard deviation values. *Abbreviations: *LBBAP, left bundle branch area pacing; RVOSP, right ventricular outflow tract septal pacing.

The fluoroscopy time for lead fixation was shorter in the LBBAP group at 2.8 (1.3–3.7) min compared to 3.1 (2–5.9) min in the RVOSP group (*P* = .02). The paced QRS duration was also significantly shorter in the LBBAP group than in the RVOSP group at 123.77 ± 10.25 versus 159.79 ± 17.0 ms (*P* = .001) **(Table 2, Figure 1)**.

In the LBBAP group, the average LVAT was 86.25 ± 17.33 ms. Within this group, LVSP was diagnosed in 32% of cases and LBBP was diagnosed in 68% of cases. Furthermore, proximal LBB capture was observed in 7% of patients, LPFP was observed in 47% of patients, LAFP was observed in 10% of patients, and LSFP was observed in 4% of patients.

There were no significant differences in the following pacing parameters between the two groups: R-wave sensing (9.6 ± 5.2 vs. 9.1 ± 4.7 mV; *P* = .91), bipolar impedance (685.9 ± 151.8 vs. 686.5 ± 158.6 Ω; *P* = .98), or pacing threshold (0.70 ± 0.29 vs. 0.64 ± 0.26 V @ 0.4 ms; *P* = .63) **(Table 2)**.

At the 6-month follow-up, the complication rates were similar in both groups. Ventricular lead revision was required in two patients, one from each group (*P* = .09); one event was due to lead dislodgment in the RVOSP group, while the other was attributed to a capture threshold increase of >2 V at 0.4 ms in the LBBAP group.

**Figure 2:**
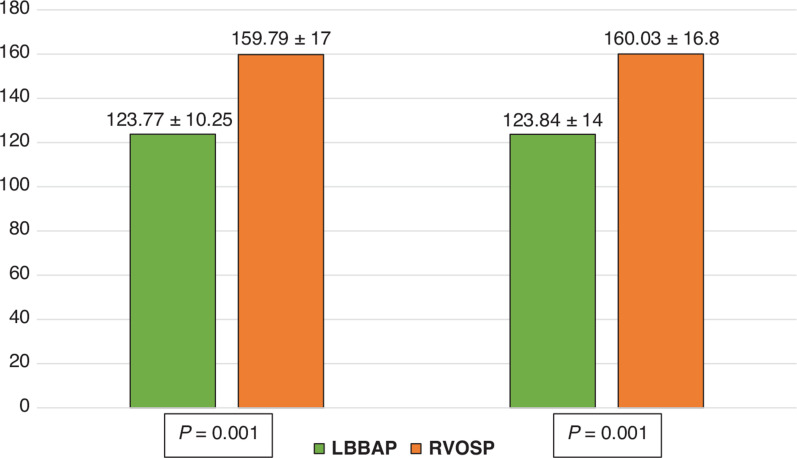
Acute and 6-month follow-up paced QRS duration (ms). The QRS duration (ms) is presented using mean ± standard deviation values within each diagram. *Abbreviations:* LBBAP, left bundle branch area pacing; RVOSP, right ventricular outflow tract septal pacing.

The paced QRS duration remained stable at the 6-month follow-up and was significantly narrower in the LBBAP group as well: 123.74 ± 13.7 versus 160.03 ± 11.6 ms (*P* < .001) **(Figure 2)**.

### The learning curve in left bundle branch area pacing

**Figure 3:**
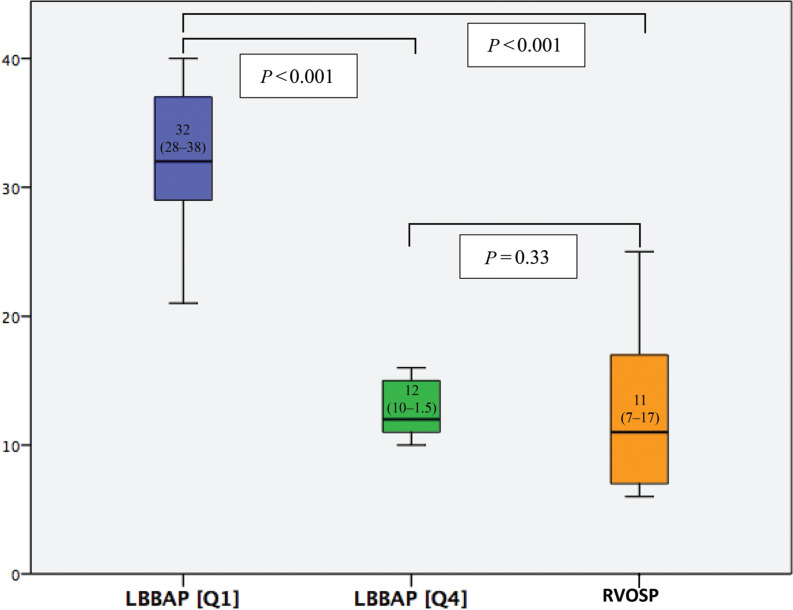
Comparison of the procedure time (min) between different left bundle branch area pacing stages (Q1–Q4) and right ventricular outflow tract septal pacing. *Abbreviations:* LBBAP, left bundle branch area pacing; RVOSP, right ventricular outflow tract septal pacing.

**Figure 4:**
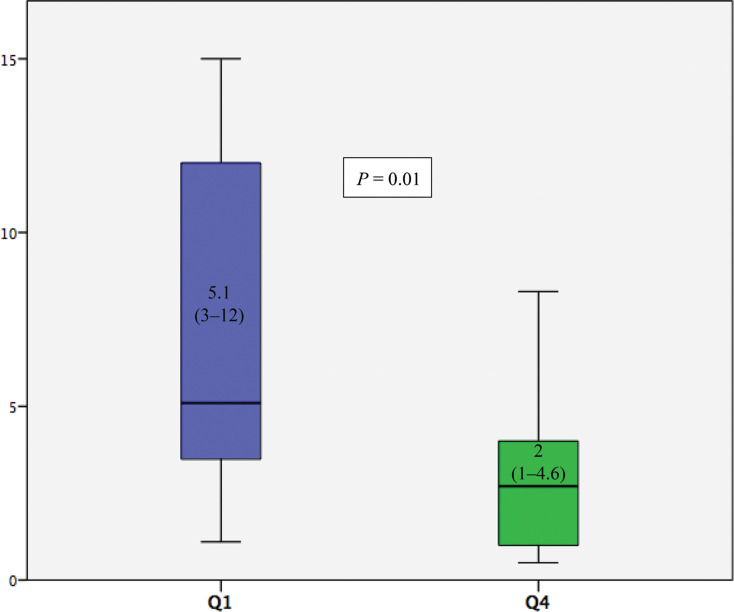
Comparison of the fluoroscopy time (min) for lead placement in left bundle branch area pacing between Q1 and Q4. The results are shown using mean ± standard deviation values within each diagram.

Regarding the learning curve for LBBAP ventricular lead placement, a comparison between Q1 and Q4 of procedures showed a reduction in the procedure time from 32 (28.5–38.5) min in Q1 to 12 (10.5–15) min in Q4 (*P* < .001) **(Figure 3, Table 3)**. There was also a significant reduction in the fluoroscopy time from 5.1 (3.4–12) in Q1 to 2.0 (1–4.6) min in Q4 (*P* < .01) **(Figure 4, Table 3)**.

**Table 3:** Learning Curve for Left Bundle Branch Area Pacing

Groups	First Quartile (Q1)	Last Quartile (Q4)	*P*
Procedure time, min	32 (28.5–38.5)	12 (10.5–15)	<.001
Fluoroscopy time, min	5.1 (3.4–12)	2 (1–4.6)	.01

Results are shown as median (Q1–Q3) values.

When comparing Q1 with the RVOSP group, we observed that the procedure time was longer in Q1 (32 [28.5–38.5] vs. 11 [7–17] min; *P* < .001). However, the procedure time was similar between Q4 and the RVOSP group (12 [10.5–15] vs. 11 [7–17] min; *P* = .33) **(Figure 3)**. No significant differences were observed in the success or complication rates between Q1 and Q4, which were 82% versus 88% (*P* = .05) and 4% versus 0%, respectively (*P* = .65).

## Discussion

LBBAP has emerged as a novel conduction system pacing technique that could overcome many of the limitations associated with HBP.^20,21^ The present study aimed to evaluate the feasibility, safety, and procedure time of LBBAP compared to RVOSP in patients undergoing pacemaker implantation, with mid-term follow-up, and to describe the learning curve for LBBAP. The primary findings of our study are as follows: (1) LBBAP achieved a high success rate comparable to that of RVOSP; (2) no serious complications were observed from this new procedure throughout the study; (3) pacing thresholds were stable and low both at the procedure and over the 6-month follow-up period; (4) paced QRS duration was shorter in LBBAP; (5) LBBAP demonstrated a rapid learning curve, with significant reductions in fluoroscopy and procedure times from the first to the last quartile; and (6) after the learning curve, the procedure time for LBBAP was similar to that for RVOSP.

This is the first study to compare outcomes between LBBAP and RVOSP. While large series have been published comparing LBBAP with RVP (including both apex and/or septal pacing^13,14,22,23^), none to date have compared it with RVOSP. Some studies have evaluated the benefits of pacing the RVOSP compared to RVAP, with a few showing long-term benefits and others demonstrating benefits only in the acute phase.^24^

Recently, the largest multicenter observational study on LBBAP outcomes, which included 2,533 patients, was published and concluded that the technique is feasible for both bradyarrhythmia and heart failure indications.^19^ This study found that the learning curve plateaued after 110 patients, consistent with our results and those in previous publications.^14^

Regarding the fluoroscopy time in our series, the median time was shorter for LBBAP compared to RVP, although the reduction of 0.3 min (2.8 vs. 3.1 min) may have limited clinical relevance. Our results align with those of other studies on LBBAP.^11,13,14,22,24,25^ This is an advantage over HBP, as the latter shows longer fluoroscopy times, ranging from 13–18 min.^26,27^ Given the operators’ experience in zero-fluoroscopy cardiac ablations and the harmful effects of ionizing radiation,^28^ every procedure in this study aimed to minimize X-ray exposure, adhering to the ALARA principle.^29^ Fluoroscopy was mainly used to check the sheath’s position before perforation and for sheath removal. In most cases, electrode penetration into the septum was mainly monitored through ECG changes, intracavitary electrograms, and impedance drop values. We believe it is important to take advantage of these records to minimize radiation exposure.

LBBAP has a longer procedure time compared to RVOSP (18 vs. 11 min). This is likely because our study includes the learning curve for this technique. These results are also consistent with those of other series.^11–13,25^

The current study demonstrated a shorter paced QRS duration in LBBAP compared to RVOSP, suggesting that it causes a better electrical synchronization of the ventricles. Our results are similar to others that have compared LBBAP to traditional RVP,^13,23,30^ with no differences between the two groups in terms of pacing threshold or adverse events during follow-up.

### Procedure and fluoroscopy time of left bundle branch area pacing: the learning curve

In order to analyze the LBBAP learning curve, we performed a comparison between Q1 and Q4. Previous investigators have reported inconsistent procedure and fluoroscopy times in LBBAP, which could be influenced by different definitions that may lead to diverse records.^11–13,25,31^ Some of them record just the time to perform the ventricular lead implant and others record the time for the whole implant.

In our study, the median fluoroscopy time significantly decreased from 5.1 min in Q1 to 2 min in Q4, which we consider noteworthy despite our relatively small cohort. Additionally, the procedure time was reduced substantially from 32 min in Q1 to 12 min in Q4. Notably, the last LBBAP quartile and RVOSP group have similar procedure times (12 vs. 11 min). Only two other studies have published comparable results regarding the LBBAP learning curve.^14,19^

We performed the single-lead technique for every implant. At the beginning of the study, we located the His bundle signal as the first step, but we realized that this often took longer than fixing the LBBAP itself. Consequently, we started progressing the sheath directly to the target area and looking for the “W” pattern in V1. We believe that the procedure and fluoroscopy times are highly influenced by the operator’s experience and performing this novel technique routinely on several consecutive patients (at least 50) can help the operator to become more proficient and promote a wider acceptance of the technique. With experience, the fluoroscopy time could be reduced to as little as 2 min and the lead placement time reduced to 12 min.

The success and complication rates were similar in Q1 and Q4, in line with other studies.^11,12^ We believe this technique can be both successful and safe from the start of the learning curve for operators experienced with pacemaker implantation. Septal perforation represents the more common acute complication associated with this technique.^31^ It is easily recognizable by a drop in unipolar impedance (<450 Ω) and a reduction in the R-wave amplitude (<1.5 mV), but it is considered a minor complication because the lead can be repositioned in the same procedure without additional risk.

The current study reinforces the effectiveness of the LBBAP technique compared to RVOSP and is in line with recent publications, displaying the same success and complication rates and comparable pacing parameters. However, long-term safety and clinical outcomes of LBBAP compared to traditional pacing remain unknown and should be investigated further in randomized clinical trials.

### Study limitations

This was a non-randomized, single-center, observational study, with the inherent limitations of this type of study given its observational nature. The limited number of patients included as well as the lack of a clinical outcome due to its short follow-up prevents us from drawing conclusions about the clinical impact of LBBAP compared to RVOSP or its prognostic impact, as it was not designed for that purpose.

Longer studies with more patients are needed to determine whether LBBAP offers clinical and prognostic improvements in patients requiring a pacemaker. Additionally, because the study was conducted only by two highly experienced operators in low-fluoroscopy procedures, results regarding fluoroscopy times may not be generalized.

## Conclusions

LBBAP is a novel physiological pacing technique that achieves a narrower paced QRS compared to RVOSP, with a high success rate and a similar complication rate. Although LBBAP prompts a significant reduction in the fluoroscopy time, the clinical relevance of this reduction is minimal. The technique has a rapid learning curve, as evidenced by the similar procedure times between the last quartile of LBBAP cases and the RVOSP group.
